# Yellow Fever Considered in Its Historical, Pathological, Etiological and Therapeutical Relations, Including a Sketch of the Disease as It Has Occurred in Philadelphia from 1699 to 1854, with an Examination of the Connections between It and the Fevers Known under the Same Name in Other Parts of the Temperate as Well as in Tropical Regions

**Published:** 1855-12

**Authors:** 


Yellow Fever considered in its Historical, Pathological, Etiological and Therapeutical relations, including a sketch of the disease as it has occurred in Philadelphia from 1699 to 1854, with an examination of the connections between it and the Fevers known under the same name in other parts of the temperate as well as in tropical regions. By R· LA ROCHE, M. D., &c., &c., in two volumes. Philadelphia: Blanchard & Lea, 1855.

DR. LA ROCHE has added another to the list of medical works that shall do honor to the literature of the profession in America.

Yellow Fever is attracting every year more and more the attention of Northern Physicians. In the Eastern Continent this scourge has been mostly confined to the intra tropical regions. On this side of the Atlantic it has not only crept along the shores of the Mexican Gulf, but has invaded several of the more Northern cities. In the Southern portions of the United States it is endemic and we know no reason why Chicago and the other cities of the Lakes may not be subject to it as an epidemic, as well as New York and Philadelphia.

We know of no man in America better qualified to do justice to the subject than Dr. La Roche.

A Review of the work is impossible, but we advise all interested in the study of the Fevers of the South and West especially to purchase and peruse it.

For sale by Keen & Lea. J.

